# The repercussions of digital bullying on social media users

**DOI:** 10.3389/fpsyg.2023.1280757

**Published:** 2023-11-21

**Authors:** Ghada A. R. Al-Turif, Hessa A. R. Al-Sanad

**Affiliations:** Department of Social Planning, College of Social Work, Princess Nourah bint Abdulrahman University, Riyadh, Saudi Arabia

**Keywords:** repercussions, bullying, digital bullying, social media, social media users

## Abstract

**Objective:**

This study aims to examine the repercussions of digital bullying on social media users, especially among university students in Saudi Arabia.

**Methods:**

It adopts a descriptive approach based on a social survey method with a sample of 640 male and female students from selected universities. A questionnaire was used to collect the data and to measure the repercussions of digital bullying on the victims, their families, and the society.

**Results:**

The findings reveal that most of the respondents agree that digital bullying has negative consequences for all the stakeholders involved. The results also indicate that female students are more aware of the repercussions of digital bullying than male students.

**Conclusion:**

The study recommends enhancing public awareness through organizing conferences, seminars, and workshops on the issue of digital bullying, and implementing and enforcing strict laws and penalties to punish the perpetrators and to prevent and reduce the harms of digital bullying.

## 1 Introduction

The field of information and communication technology has undergone rapid and significant changes, especially with the advent of the information revolution. This has led to various transformations in the economic, political, and social spheres, as information can be transmitted quickly and accurately across different domains (Kumar and Khare, 2022). It has also enabled a wide range of communication and interaction opportunities through the creation of social networks and online platforms, which have become popular among various segments of society, especially the youth. These platforms have also influenced the cultural and cognitive aspects of individuals and communities.

Digital bullying is a widespread issue that has a negative impact on the social media ecosystem (John et al., 2018). Although the terms are frequently used interchangeably, it is important to remember that cyberbullying and digital bullying refer to different forms of online abuse. Cyberbullying is a type of premeditated and targeted abuse that generally focuses on chosen targets (Raskauskas and Huynh, 2015). Digital bullying, on the other hand, refers to a broader spectrum of unpleasant online activities. These activities include a variety of undesirable behavior such as harassment, defamation, the distribution of false information, and even impersonation (Chun et al., 2020). The aforementioned minor distinction underscores how profound and intricate the issues that users of social networking sites confront. Understanding the many types of harm experienced by people in the constantly increasing realm of online contacts in the present atmosphere necessitates exploring the distinctions between cyberbullying and digital bullying.

Social networks have many benefits for both individuals and society, as they facilitate the exchange of culture and knowledge, the access to information, and the use of educational and professional resources. They also play a role in social interaction, job creation, marketing, and trade, as well as forming virtual relationships that replace conventional social ties within a virtual social system that consists of individuals, groups, and organizations that share various personal, social, psychological, ideological, religious, and other factors (Abdulrahman, 2013).

However, social networks also have some drawbacks and risks, as they can be mis-used for destructive, abusive, and violent purposes, such as cybercrime, hacking, terror-ism, addiction, rumormongering, social isolation, psychological problems, virtual communication illusion, privacy violations, and so on (Zaidi and Banai, 2022). One of the negative phenomena that occurs on social media is digital bullying, which is the focus of this study.

Despite the plethora of academic research and literature that has investigated many elements of social networks and the accompanying risks, there is an urgent need for extensive investigations into the specific topic of cyberbullying. Previous research, which has made significant contributions, has explained the larger difficulties faced by social networks (Ioannou et al., 2018; Chan et al., 2021; Ademiluyi et al., 2022). However, in the contemporary research environment, it is critical to pay special attention to the complex dynamics, fundamental causes, and viable responses to cyberbullying.

The investigation of digital bullying focuses mostly on social media users, as these platforms are the primary venues for the incidence of such abuse. By focusing on this specific demography, it is possible to apply accurate and personalized therapies (Abarna et al., 2023). Given the many dynamics and hazards connected with various online activities, including all internet users in the area of study may possibly reduce the efficacy of efforts. Digital bullying is a unique and dynamic problem that needs specialist solutions and methods to protect people’s well-being, particularly young people who use social media extensively (Lamba et al., 2016; Chen and Luppicini, 2021; Nasla et al., 2021).

The necessity to address challenges peculiar to Saudi Arabia’s environment prompted experts to investigate the phenomenon of cyberbullying in the country. The Kingdom of Saudi Arabia has swiftly embraced information and communication technology (ICT), with both positive and bad consequences (Alzahrani, 2020). Digital bullying has become a major cause of worry, negatively impacting those who utilise social media platforms in unique and significant ways (Fati, 2021). The study intends to investigate the specific issues experienced by media users in that nation in order to fill a research gap and provide informative information that may assist build interventions to address the problem of cyberbullying in the Saudi Arabian setting.

The current study aims to provide a more comprehensive and in-depth examination of digital bullying, hence addressing the complexities underlying these prevalent phenomena. While previous research has established a foundation for understanding the whole range of risks connected with social networks, the current study intends to focus primarily on digital bullying (Jabeen and Treur, 2018). The study’s purpose is to make a substantial contribution to the establishment of preventative measures, welfare programmes, and regulations that can effectively reduce the negative impacts of cyberbullying. The objective of this research is to create a more secure and productive environment for everyone who uses the internet.

### 1.1 Study problem

The phenomenon of bullying has traditionally involved verbal, physical, or social forms of aggression, such as name-calling, beating, or isolating someone from social activities. However, with the advancement of information and communication technology and the emergence of cyberspace and various online platforms that form a large-scale virtual community for many people (such as Facebook, Twitter, Instagram, Telegram, and chat rooms), a new type of bullying has arisen, which is digital bullying. This is the most recent form of bullying that relies on technological means, and thus the confrontation be-tween the bully and the victim has shifted from face-to-face to online in a virtual environment. Since the online space has no geographical boundaries for its participants, it is possible for someone to receive abuse beyond their real community and to experience it in the virtual world. Digital bullying often undermines the dignity of users in a publicly visible way (Patchin and Hinduja, 2017), and other participants can join in the abuse and ridicule by responding to and engaging with the offensive content through negative comments and reposts (Doll et al., 2017).

According to statistics from the United Nations Children’s Fund (UNICEF), 90% of young people around the world have experienced some form of bullying, either psycho-logical or physical (Al-Qaddouri and Abdelkader, 2020). Scientific research also suggests that 7 out of 10 young people have been exposed to online abuse at some point for psychological and social reasons against others with certain characteristics such as race, religion, people with special needs, etc., (Abu Ghazaleh, 2018). Everyone in society has the right to live with equal rights with others without discrimination (Beran, 2018), and it is important to encourage young people to express themselves freely with-out harming others and to promote digital citizenship to contribute to a global society (Cook et al., 2019).

Therefore, digital bullying has attracted great attention from researchers in the educational, psychological, and social fields due to its increase and spread in recent decades due to technological progress and the growing use of young people for various modern technology tools and social media applications. This has resulted in the reproduction of bullying through the online space, which is one of the negative phenomena that entails many negative impacts on various segments of society, whether psychologically, emotionally, socially, or academically (Pentan and Al-Asmar, 2019). It also affects not only the bully and the victim but also extends to the victim’s family and society as a whole. Bullying can change the behavior of the individual victim from a normal person to a person with behavioral deviation to defend against the bullying shown by others towards him. It also reflects on his personal and social life and affects his interaction and family and social relations as well as the achievement of the goals of the group and society alike. Therefore, it is important to study it in a scientific manner to reach proposals that aim to address and reduce it (Al-Sayed, 2020).

Hence, the problem of this research lies in identifying the impacts of digital bullying on social media users by answering the following questions:

1. What are the forms of digital bullying among social media users?
2. What are the causes of digital bullying from the perspective of social media users?
3. What are the impacts of digital bullying on victims, their families, and society?
4. Do respondents’ attitudes towards digital bullying differ according to gender and monthly income?

### 1.2 The objectives of the study

This study seeks to explore the repercussions of digital bullying on social media users (victims, their families and society) and to propose solutions that can mitigate the negative impacts on the victim, his or her family and society.

### 1.3 The importance of the study

- Digital bullying is a pressing issue at the local, regional, and global levels that demands attention due to its effects on all segments of society, especially the youth.
- The study contributes to the knowledge base on digital bullying through social media platforms by enriching the Arab literature with information about digital bullying in the Saudi context.
- This study is important because it focuses on the youth category, which is a crucial stage for developing social, physical, and psychological skills that are essential for the formation of human personality. They are also the main force in building societies, so it is vital to understand and address the challenges they face.
- The findings of the study are beneficial for policymakers to identify the motives and causes of the rise of digital bullying and its adverse effects on victims, their families and society as a whole, and to devise strategies that can tackle this problem.

### 1.4 Theoretical framework

Digital development has brought rapid advances in technological means, but this progress achieved by information technology in recent decades has also brought multiple risks and challenges, including digital bullying (Halachová, 2014). This has become one of the most serious threats to young people in the digital space and raises many concerns in society (Cheng et al., 2018).

The research is based on a wide theoretical framework that asserts that a range of elements, such as social, psychological, economic, and technological variables, shape digital bullying, particularly in the setting of social media. The issue is caused by the rapid growth and complete integration of information and communication technology (ICT). This theoretical position contends that the prevalence of cyberbullying is influenced not just by individual characteristics, but also by broader cultural, economic, and technological factors. This research investigates the complicated roots of cyberbullying, the different forms it may take, and the far-reaching consequences it has on the targets, their families, and society as a whole.

#### 1.4.1 Characteristics of digital bullying

Digital bullying differs from other forms of bullying in the degree of danger it poses, because the bully can erase the trace and hide his or her identity. The bullies often use fake names and identities and therefore cannot be verified, which encourages them to continue digital bullying without fear. Moreover, the bullies do not see the direct impact of the harm they cause, which does not deter them from bullying or make them feel emotional remorse (Guan et al., 2016). Digital bullying can also occur anywhere, and a bully can reach a vast number of audiences and access and exploit the information of targeted victims (Zubaidi and Tariq, 2020).

#### 1.4.2 Forms of digital bullying

Due to the development and expansion of the use of the Internet in various transactions, there are multiple forms and types of digital bullying, which are as follows:

- Electronic harassment: sending abusive and insulting messages or threatening messages *via* email and repeating it.
- Defamation: sending false information or spreading rumors about a person with the aim of harming him or her.
- Impersonation: hacking someone’s personal account and pretending to be him or her to send or post electronic materials to trap or discredit this person.
- Disclosure: sharing someone’s secrets, embarrassing information about him or her, or pictures and posting them on the internet.
- Deception: tricking someone into revealing his or her secrets, embarrassing information about him or her, or pictures in a situation that others do not want to see and then posting them on the Internet or sending them to others.
- Harassment and extortion: sending nasty and insulting messages repeatedly through multiple electronic communication channels to create intense fear in the other party.
- Exclusion: deliberately and cruelly excluding someone from an online group (Aboulela, 2017).

#### 1.4.3 Causes of digital bullying

The phenomenon of digital bullying is attributed to a set of causes, which we review as follows:

- Social causes: disruption of social and family relations digital bullying may result from the presence of deficiencies or defects in the family structure, such as family disintegration problems, which take various forms such as separation, divorce, continuous disputes, the absence of one of the parents from the family, parents’ ignorance of socialization methods, harsh treatment, violence within the home, and the inability to control the behavior of children (Amer, 2019). Frequent exposure to physical harm and harsh treatment in the home leads to children’s tendency towards deviant and delinquent behavior (Hinduja and Patchin, 2006).
- Psychological causes: psychological factors play a major role in shaping bullying behaviors, as they may result from subjective motives stemming from the bully’s personality, which is characterized by aggression and authoritarianism, or a strong physical structure that pushes him or her to show his or her strength, irritability, recklessness and weakness of religious scruples, the desire to surpass the complexities of technical means, passion for collecting and seizing information, envy, jealousy and the desire to take revenge on others (Abu Ali, 2018). In addition to behavioral disorders and the imbalance of the personal structure of the individual and psychological, organic and mental diseases that generally affect the human personality and behaviors because of the mental, psychological or functional disorders that occur in the individual, and this means that there is a relationship between normal and deviant behavior and the health and psychological state of the individual (Al-Tarif, 2012).
- Economic causes: economic conditions play a role in the occurrence of bullying as the bully may feel empowered and in control due to the high economic level. The opposite is also possible, as perhaps his or her belonging to a poor class and the material need causes him or her a sense of inferiority, frustration and weakness, so he or she practices bullying to vent his or her feelings (Abdulaziz, 2017).
- Media and technological causes: the scientific, technological and cognitive revolution has led to the ease of browsing social networking sites by children, adolescents and adults, watching conflicts, quarrels and videos that contain many scenes of violence, watching horror movies, and the spread of electronic games that contain many scenes of violence and abuse and trying to imitate them in their real lives (Al-Ammar, 2016). This implies that violence is normalized as a way of claiming rights. A study supports this claim (Mohammed, 2012). However, there are also technical motives, such as the desire to show technical superiority and the passion for collecting and accessing in-formation. Moreover, there is a lack of information security awareness programs to pre-vent digital bullying, and the laws that deter it are either weak or poorly enforced.

#### 1.4.4 The repercussions of digital bullying

Digital bullying is closely related to everything that occurs on social media platforms, whether through their content or through the communication technologies they use. The language of violence has become the dominant mode of communication and interaction among young people with their friends and others, which ultimately undermines the social network and causes various social and psychological problems that affect not only the victim, but also the victim’s family and society as a whole. This has an impact on the security system of society (Zaidi and Banai, 2022). We will discuss the repercussions of digital bullying on the bully, the victim, their family, and society as follows:

- The repercussions of digital bullying on the victim: bullying has multiple negative consequences for victims, as it may lead to psycho-logical, emotional, and behavioral problems in the long term, such as depression, loneliness, isolation, anxiety, addiction and self-harm. The victim becomes ostracized and un-wanted, in addition to low academic achievement due to dropping out of school or frequent absenteeism or escaping from school out of fear or distress. Furthermore, poor social relations and lack of trust in others make them more susceptible to exploitation and lacking self-assertion skills. They may also experience several psychosomatic symptoms such as headache and abdominal pain. Some may resort to suicide as a way to escape from their suffering. Repeated bullying has long-term adverse effects on victims that last for years. Victims of bullying in their early years are more prone to depression and low self-esteem compared to their peers who have not been bullied (Penis, 2020). The victim may also adopt aggressive behavior and bullying as a result of their exposure to it. The victim’s withdrawal from social activities in their social environment may increase until they be-come silent and isolated. They may resort to suicide, as studies have shown that suicide victims are constantly increasing due to bullying (Mohammad, 2020).
- The repercussions of bullying on bullies: bullying is not only an isolating behavior by its perpetrators, but also part of an anti-social pattern that breaks or weakens the rules that govern it. Bullies are willing to engage in unacceptable social behavior such as assaulting other people’s property, shoplifting, skipping school, and frequent drug use. The effects of bullying on bullies can be presented as outcomes of their behavior in the following points: denial of education, expulsion from school, drug addiction, aggression and involvement in criminal acts, legal violations, constant conflicts with others, vandalism and dropping out of school, and early sexual deviations (Mohammad, 2019).
- The repercussions of digital bullying on the victim’s families: the phenomenon of bullying has many negative impacts on the families of the victims, as they suffer from the consequences of their child being exposed to bullying behavior that affects their health, psychological state and social relations in their family, friends, and society. Sherri (2018) identifies many adverse effects on the victim’s family, such as: the parents’ feeling of helplessness to remedy or improve the situation, feeling lonely and isolated, being preoccupied with the circumstances that their child is going through, and neglecting their health, feeling sad, feeling of failure due to their inability to protect their bullied child.

The family has a vital role in preventing digital bullying by teaching children how to defend themselves, following up and listening to them, supporting them, and working to build a strong personality for them, enhancing their self-confidence and dealing wisely and firmly when they are exposed to digital bullying, and striving to achieve the safe use of social media. The family also plays a role in raising children on human values and morals such as tolerance, equality, respect, love, helping the weak, and others. And ensuring the monitoring of the different behaviors of children at an early age and finding and correcting the wrong behaviors. The family can also take part actively in combating bullying by volunteering with local community institutions that are interested in combating digital bullying through programs and activities that contribute to raising awareness of the phenomenon, its negative effects, and ways to address it.

- The repercussions of digital bullying on society: the repercussions of digital bullying are not confined to victims and their families but extend to society as well. It may cause a disruption in social relations, the spread of hatred and animosity among members of society, and difficulty in social adaptation in school, work, or social environment in general. It also leads to an increase in social problems such as the problems facing security and educational institutions such as academic delay, escaping from school, drug addiction and deviating from social values and norms, regulations and laws through delinquency, crime, and suicide, which require allocating a budget to address and prevent these problems (Zaidi and Banai, 2022).

The practice of aggression towards public property has clear economic effects as well as social ones. It leads to the waste of public money and delays the development plans that the state is pursuing to develop society and its facilities in various economic and developmental fields. But when these plans meet obstacles that hinder progress, the eco-nomic impact becomes evident. The delay of plans financially and temporally is followed by the delay of services that benefit members of society, due to the tendency to repair the damage caused by aggressive behavior on public facilities such as roads, schools, entertainment places etc., which requires harnessing budgets and efforts to address the negative effects that have been reflected on society (Abu Ali, 2018).

### 1.5 Literature review

The repercussions and effects of digital bullying on social media users have been explored by various researchers in different contexts. This section will review some of the relevant studies and provide a critical commentary on them. The studies include:

Qutb (2022) conducted a social survey to examine the concept of digital bullying among Saudi women at the undergraduate level, using a questionnaire for 788 participants. The study revealed that the main motives and social causes of digital bullying were related to the external appearance and the personal content that women shared on social networks. The study suggested that awareness campaigns, legal sanctions, religious values and self-regulation were important factors to prevent digital bullying. Moirs and Mehrezi (2022) investigated the nature, forms and impacts of Digital bullying, finding that the most common types of bullying were harassment, defamation, identity theft, disclosure of secrets, deception, exclusion and electronic stalking. The study also re-ported that Digital bullying led to various psychological, emotional and behavioral problems for the victims, such as depression, loneliness, introversion, anxiety, addiction, self-harm or suicide. Moreover, Digital bullying affected the victims’ trust in others, social relationships, participation in social activities, school attendance, aca-demic performance and aggression levels. The study proposed several strategies to reduce the prevalence of this phenomenon.

Mahmoud (2021) explored the emergence of Digital bullying and identified the groups most vulnerable to bullying, such as children with disabilities, learning difficulties, introversion or physical differences. The study indicated that Digital bullying had negative effects on the victims, such as spreading rumors, exclusion from the group, psychological problems like frustration, depression and psychosomatic symptoms that could lead to suicide. The study also discussed the causes of bullying, such as emotional deprivation, parental neglect, violent imitation or repression in the environment. The study recommended the importance of designing appropriate pro-grams and guiding parents on how to deal with children (Mahmoud, 2021). Ben Dada and Karim (2021) examined the manifestations of Digital bullying among university students, showing that there were five forms of Digital bullying among this group: exclusion, sexual harassment, inconvenience and privacy violation, insult and threat, and mockery and distortion. The study emphasized the need for developing preventive programs to reduce this phenomenon because of its serious psychological and social effects on the individual and society.

Dalaala and Maghouni (2021) conducted a study to explore the electronic traffic through social media, the role of gender and the time spent by the individual on social media, in influencing the bullying behavior. The study found a statistically significant relationship between the gender variable and the rates of Digital bullying, but no statistically significant relationship between the time spent by the individual on social media and the increase in the rate of bullying. The study recommended the importance of raising the awareness of young people to prevent Digital bullying (Dalaala and Maghouni, 2021). Ben Salem (2020) aimed to identify the level of students’ awareness of the psychological effects of Digital bullying. The study used a descriptive approach and a questionnaire for 150 undergraduate students. The study reported that Digital bullying caused depression, social anxiety, low self-worth, feelings of psychological distress, anger at the aggressor, and serious social problems for the victims. The study suggested proactive strategies to counter bullying.

Mohammed (2020) explored the causes and factors that lead to bullying and the definition of its forms and effects. The study was a descriptive study and used a questionnaire for 242 pre-university students. The study revealed that the causes of Digital bullying were related to parents not monitoring their children’s devices, violent electronic games, violent cartoon films, domestic and community violence, family dysfunction. The study also indicated that the forms of bullying were multiple chat rooms, video watching, phone call, instant messaging, photos, email, impersonation, exclusion or cyber ostracism. The study also reported that the risks of Digital bullying were a sense of fatigue and exhaustion, lack of concentration in studying, feeling up-set, lack of sleep. Zayed (2020) examined the extent to which adolescents were exposed to Digital bullying. The study was a descriptive study and used a survey methodology and a questionnaire for 300 secondary school students. The study showed that the most common forms of Digital bullying that adolescents faced through digital media were the dissemination of personal secrets, opinions and beliefs, temptation to engage in in-appropriate behavior and threat to spread it, threat through digital media, misuse and dissemination of personal photos and videos, sharing an inappropriate video, logging into the personal account and publishing private matters, and receiving in-appropriate text messages from strangers.

Al-Baris et al. (2019) investigated the level of Digital bullying and exposure to it from the victim’s point of view. The study found that Digital bullying was one of the most prevalent behaviors in this era, and it had profoundly serious psychological and social problems with negative consequences on the cognitive, social and emotional development of bullies and victims. Mohammad (2019) conducted a study to identify the reality and forms of Digital bullying among students in the secondary stage, using a descriptive approach and a questionnaire for 132 male students and 127 female students. The study revealed that the most prominent forms of bullying were ridicule, defamation, spreading rumors, publishing disturbing images, harassment, insults, repeated abuse, impersonation, identity theft, disclosure of secrets and electronic stalking. Abu Ali (2018) aimed to identify the social and cultural dimensions of the phenomenon of bullying in secondary schools. The study used a questionnaire for 250 male secondary school students. The study indicated that the phenomenon of bullying was not limited to males only, but also included females. The study also reported that the reasons for the spread of the phenomenon of bullying were social, economic, cultural, and psychological (Mohammad, 2019).

The literature on the study of digital bullying at various levels shows that most of the studies focused on the forms, types and causes of Digital bullying, and some studies dealt with the repercussions of digital bullying on the victim and the bully. The current study is different from previous studies by addressing the repercussions of digital bullying on the victim’s family and society as a whole.

The current study is similar to previous studies in the nature of the methodology used, which is the descriptive approach, and the tool used to collect data and information, which is the questionnaire. The current study is also similar to some previous studies in the sample used to apply the questionnaire to undergraduate students. The current study has benefited from previous studies in forming a comprehensive understanding of digital bullying in general and its repercussions in particular and using the results and recommendations of the studies in writing the theoretical framework for the research, determining the methodology, building the study tool, discussing the results, and enriching the study with references, books, studies and scientific journals.

## 2 Materials and methods

### 2.1 Type of research

The current study is a descriptive and analytical study that aims to identify the re-percussions of digital bullying on social media users. Descriptive studies provide information and facts about the reality of the current phenomenon, clarify the relationship be-tween different phenomena and help predict the future of the phenomenon itself (Pandey, 2014). The study also uses the quantitative approach (questionnaire) to collect and describe data numerically and present the results, as well as to extract conclusions, generalizations, and new relationships. The study also reviews previous literature and collects and analyzes data to identify the repercussions of digital bullying on social media users, and then performs statistical processing, analysis, and discussion of the results.

### 2.2 Method

The study relies on the social survey methodology as one of the main methods used in descriptive analytical studies, which depends on collecting data on a particular phenomenon and analyzing that data to reach results. The social survey focuses on the study of social problems and phenomena, as it covers all aspects of our social life (Al-Tarif, 2019). The study uses the questionnaire to collect data from the sample, as it is one of the most appropriate methods for the nature of this study because it helps in describing the phenomenon under study by providing the necessary data for that. It also enables the researcher to study a small sample of the population and generalize its results to all members of the community concerned with the study (Al-Maaytah, 2011). The sample survey method is one of the most used methods in social research because it saves time, effort, and money within the limits of the capabilities of researchers, in addition to that it comes with accurate results (Hassan, 2016).

### 2.3 Limits

- Objective limits: The repercussions of digital bullying on social media users.
- Spatial boundaries: A number of universities in the Kingdom of Saudi Arabia (Imam Muhammad bin Saud Islamic University, Imam Abdulrahman bin Faisal University, Umm Al-Qura University, Hail University, Jazan University).
- Human limits: A random sample of male and female students in Saudi universities specified in the study.
- Time limits: Saudi Arabia 2022–2023.

### 2.4 Study sample

The scientific research in descriptive studies deals with a scientific phenomenon emanating from a large population, and the researcher cannot study all that population, but chooses a representative sample of it (El-Beltagi et al., 2012). To determine the representative sample, the regions of the Kingdom have been divided into five regions: north, south, east, west and center of the Kingdom. These regions can be considered as strata in the first stage (stratified sampling), and all universities in the same region are treated as clusters (cluster sampling) as a second stage. In the third stage, for each region, a random sample of universities (simple random sampling) is selected. As a final stage, a random sample is selected from each cluster, i.e., a random sample of students of both sexes in universities through simple random sampling. This ensures a high degree of randomness and representation of the selected sample of students in different regions and departments (Al-Tarif, 2012). Therefore, a random sample of male and female students was selected in five universities randomly chosen to represent each region of the Kingdom. The universities were Imam Muhammad bin Saud Islamic University, Imam Abdul Rahman bin Faisal University, Umm Al-Qura University, Hail University and Jazan University. The total sample was 640 respondents.

### 2.5 Study tools

The questionnaire was chosen as the main tool for collecting field data according to the nature and objectives of the study. The questionnaire was prepared based on the theoretical framework and previous studies in this field.

The questionnaire consists of primary data related to gender, monthly income, and 42 statements that measure three main axes:

1. Forms of digital bullying among social media users, consisting of 9 statements.
2. Causes of digital bullying from the perspective of social media users, consisting of 4 statements.
3. Repercussions of digital bullying on victims, their families and society, consisting of 29 statements distributed over three areas:

- Repercussions of digital bullying on social media users, consisting of 10 statements.
- Repercussions of digital bullying on the families of victims, consisting of 9 statements.
- Repercussions of digital bullying on society, consisting of 10 statements.

The response to the questionnaire statements was on a three-point scale according to the Likert method, with three responses: (agree, agree to some extent, disagree), and these three responses take the three scores: (3, 2, 1) respectively. To verify the reliability and validity of the questionnaire, it was applied to the members of the pilot sample consisting of 70 male and female students, and the reliability and validity of this questionnaire were calculated as follows:

#### 2.5.1 Reliability of the questionnaire

The reliability of the Digital bullying survey statements on social media users was calculated by calculating the correlation coefficients between the item scores and the over-all scores of the axis to which the item belongs. The results showed that the correlation coefficients between the score of each item and the total score of the axis to which the item belongs ranged from 0.53 to 0.91, all of which were statistically significant at the level of 0.01 ≥ α, which indicates the internal consistency and reliability of all the statements of the study questionnaire. The reliability of the axes of the questionnaire was also calculated by two methods: Cronbach’s alpha coefficient and Spearman-Brown split-half method. The results indicated that the total reliability coefficients for the first and second axes and the three areas of the third axis of the questionnaire by Cronbach’s alpha method were: 0.930, 0.727, 0.907, 0.896, 0.947 respectively, and by Spearman-Brown split-half method were: 0.955, 0.947, 0.922, 0.900, and 0.955 respectively, all of which were high, indicating the overall reliability of the questionnaire axes.

#### 2.5.2 Validity of the questionnaire

- Apparent validity: the questionnaire was presented to a group of specialized professors in the Department of Sociology and Social Work at Princess Nourah bint Abdulrahman University and Imam Muhammad bin Saud Islamic University to determine the suitability, importance, clarity, and wording of the statements. The questionnaire was revised based on their opinions and finalized.

The validity of the questionnaire statements was calculated by calculating the correlation coefficient between the score of the item and the total score of the axis to which the item belongs, after removing the score of the item from the total score of the axis. This means that the rest of the statements of the axis are used as a criterion for the item. The results indicated that the correlation coefficients between the score of each item and the total score of the axis to which the item belongs (after removing the score of the item) ranged from 0.42 to 0.88, and all of them were statistically significant at the level of 0.01 ≥ α, which indicates the validity of all the statements of the questionnaire.

- Validity of the axes: the validity of the questionnaire axes was calculated by calculating the self-validity coefficient for each axis, which is equal to the square root of the reliability coefficient by Cronbach’s alpha method. It was found that the self-validity coefficient of the questionnaire axes ranged from 0.64 to 0.89, and all of them were high, which indicates the validity of the axes of the questionnaire.

### 2.6 Statistical methods used to process data

To achieve the objectives of the study and analyze the collected data, various appropriate statistical methods were used using the statistical packages for social sciences (SPSS). A set of statistical methods were used to calculate the reliability and validity of the research tool and answer its questions, and these methods are: Cronbach’s alpha coefficient, Spearman-Brown split-half reliability coefficient, Pearson’s correlation coefficient, frequencies and percentages, means, Chi-Square test, Independent Samples Test, and one-way analysis of variance (One-Way ANOVA) followed by Least Significant Difference (LSD) test to determine the direction of statistically significant differences.

### 2.7 Life science identifiers

Life Science Identifiers (LSIDs) for ZOOBANK registered names or nomenclatural acts should be listed in the manuscript before the keywords with the following format:

urn:lsid:<Authority>:<Namespace>:<ObjectID>[:<Version>].

## 3 Results and discussion

Table 1 shows that there is a slight difference between the size of males and females in the sample, although the percentage of females is higher, reaching 56.25%, while the percentage of males reached 43.75% of the total sample size. Regarding the monthly in-come variable, more than half of the sample had middle income (59.4%), followed by low income (28.1%), while the lowest percentage had high income (12.50%) of the total sample size.

**Table 1:** Distribution of the study sample by gender and monthly income variables.

Variable	Variable subsets	Frequency	Percentage	Variable
Gender	Males	280	43.75%	Males
	Females	360	56.25%	Females
Monthly income	High	80	12.50%	High
	Medium	380	59.4%	Medium
	Low	180	28.1%	Low

Table 2 shows that there are statistically significant differences (at the level of 0.01 ≥ α) between the frequencies of the responses of the respondents in favor of the response (agree) on all statements of the first axis: (form of digital bullying among social media users). This means that the majority of respondents agreed statistically with all forms of digital bullying among social media users. The averages of the statement of the first axis ranged from 2.30 to 2.74 and all these averages fall in the range of response (agree; which ranges from 2.33 to 3), which confirms the agreement of the sample members on all forms of digital bullying among social media users, except for one form of bullying that was partially agreed, which is the item “impersonation.” The highest mean for the statements of the ax-is from the perspective of the sample was 2.74 out of 3 and was for the form of digital bullying (Hostile messages that hurt the feelings of the recipient, such as mocking appearance, name-calling, and so on), while the lowest mean for the statements of this axis was 2.30 and was for the form of digital bullying (impersonation). The results of the study are consistent with the findings of Zayed (2020), Ben Dada and Karim (2021), Moirs and Mehrezi (2022) as well as Aboulela (2017), and Mohammad (2020).

**Table 2:** Chi-square test results to examine the differences between the frequencies of the respondent’s responses to the statements of the first axis (forms of digital bullying among social media users).

#	The statement	Agree		Partially agree		Disagree		Average	*K* ^2^	Rank
		Freq.	Perc.	Freq.	Perc.	Freq.	Perc.			
1	Hostile messages that hurt the feelings of the recipient, such as mocking appearance, name-calling, and so on.	536	83.75	40	6.25	64	10.00	2.74	733.41**	1
2	Distorting reputation and spreading rumors and lies about the victim.	504	78.75	60	9.38	76	11.88	2.67	594.66**	3
3	Threatening to disclose secrets and publish personal information (extortion).	456	71.25	60	9.38	124	19.38	2.52	423.66**	6
4	Harassment and sharing of offensive content.	476	74.38	68	10.63	96	15.00	2.59	486.96**	4
5	Using video clips of a person without their permission or knowledge.	456	71.25	80	12.50	104	16.25	2.55	415.41**	5
6	Hacking, surveillance, and theft of personal accounts.	388	60.63	84	13.13	168	26.25	2.34	231.05**	7
7	Impersonation.	348	54.38	136	21.25	156	24.38	2.30	128.45**	9
8	Exclusion and expulsion from social media groups and other online groups.	388	60.63	124	19.38	128	20.00	2.41	214.55**	8
9	Endorsing bullies’ comments by reposting comments/images/clips/retweets/etc.	508	79.38	56	8.75	76	11.88	2.68	611.46**	2

*Statistically significant at the level of 0.01 ≥ α.

Table 3 shows the multifaceted factors contributing to acts of violence, encompassing social, psychological, economic, and technological dimensions. These factors include poor social relations, weak family control, exposure to violence in the home or community, mental disorders, jealousy, the desire for attention, a sense of emptiness, income levels, material deprivation, high prices, and the influence of violent media portrayals. The statistically significant difference (at the level of 0.01 ≥ α) between the frequencies of the responses of the respondents in favor of the response (agree) on all statements of the second axis: (causes of digital bullying among social media users) as shown in Table 3. The average of the statements ranged from 2.31 to 2.82 and all these averages fall within the range of response (agree; which ranges from 2.33 to 3) except for one item that was partially agree, which was for “economic reasons.” The highest mean for the statements from the perspective of the sample was 2.82 out of 3 and was for psychological reasons, followed by social reasons, then reasons related to technological development, while the lowest mean for this axis was 2.31 and was for economic reasons. The results of this study are consistent with both (Abu Ali, 2018; Mohammed, 2020; Mahmoud, 2021; Qutb, 2022) which dealt with social motives and causes for bullying.

**Table 3:** Chi-square test results to examine the differences between the frequencies of respondents’ responses to the statements of the second axis (causes of digital bullying among social media users).

#	The statement	Agree		Partially agree		Disagree		Average	*K* ^2^	Rank
		Freq.	Perc.	Freq.	Perc.	Freq.	Perc.			
1	Social reasons	516	80.63	64	10.00	60	9.38	2.71	644.16**	2
2	Psychological reasons	552	86.25	60	9.38	28	4.38	2.82	808.86**	1
3	Economic reasons	332	51.88	172	26.88	136	21.25	2.31	102.05**	4
4	Reasons related to technological development	384	60.00	136	21.25	120	18.75	2.41	205.40**	3

**Statistically significant at the level of 0.01 ≥ α.

Supplementary Table S1 shows that there are statistically significant differences (at the level of 0.01 ≥ α) between the frequencies of the responses of the sample members in favor of the response (agree) on all statements of the third axis related to the three areas: (the repercussions of digital bullying on social media users, the repercussions of digital bullying on the families of victims, and the repercussions of digital bullying on society). The mean of the statements of the third axis ranged from 2.44 to 2.83 and all these means are in the range of response (agree). The highest mean for the area of (the repercussions of digital bullying on social media users) from the perspective of the sample was 2.83 out of 3 and was for (low self-esteem and lack of confidence in oneself and others), while the lowest mean for the statements of this area was 2.44 and was for (unwillingness to practice hobbies). On the second area about (the repercussions of digital bullying on the families of victims), the mean ranged from 2.31 to 2.69 and all these means are in the range of response (agree). The highest mean for the statements of the area about (the repercussions of digital bullying on the families of victims) from the perspective of the sample was 2.69 out of 3 and was for (confusion and lack of family knowledge of how to deal with the problem), while the lowest mean for the statements of this area was 2.31 and was for (low family productivity). As for the statement of the third area about (the repercussions of digital bullying on society), the means ranged from 2.54 to 2.80 and all these means are in the range of response (agree). The highest mean for the area of (the repercussions of digital bullying on society) from the perspective of the sample was 2.80 out of 3 and was for (the emergence of bullying and hostile personalities against society), while the lowest mean for the statements of this axis was 2.54 and was for (the spread of a culture of violence as acceptable solutions to social problems). The results of the repercussions of digital bullying on social media users, victims’ families, and society are consistent with the findings of Al-Baris et al. (2019), Ben Salem (2020), Mahmoud (2021), as well as Mohammad (2020) on the various effects of digital bullying on the social and psychological aspects.

To answer the fifth question: do respondents’ attitudes towards digital bullying differ according to gender and monthly income? The Independent Samples Test was used to examine gender differences, and One-Way ANOVA analysis followed by LSD test to determine the direction of statistically significant differences. Table 4 shows that there is a statistically significant difference (at the level of 0.05 ≥ α) between the mean of males and females in: (form of digital bullying among social media users, causes of digital bullying from the perspective of social media users), (the repercussions of digital bullying on social media users), (the repercussions of digital bullying on society) due to the gender variable, as the values of F are statistically significant in favor of the female mean in all cases. We find that female members of the sample of social media users are more aware of the form and causes of digital bullying among social media users, as well as more aware of the repercussions of digital bullying on both social media users and society, compared to males. This result is consistent with (Dalaala and Maghouni, 2021) that indicates a relationship between gender and Digital bullying. The results also showed that there were no statistically significant differences in each of the (repercussions of digital bullying on the families of victims) due to the gender variable, as the value of T is not statistically significant. This indicates that respondents’ attitudes towards digital bullying related to the re-percussions of digital bullying on the families of victims do not differ according to gender.

**Table 4:** Results of the Independent Samples Test to examine the different attitudes of respondents towards digital bullying according to gender.

Gender	Count	Dependent variables	Average	Standard deviation	Degrees of freedom	Value (T)	Significance level
Males	280	Forms of digital bullying among social media users.	2.44	0.64	638	3.34	0.01**
Females	360		2.60	0.53			
Males	280	Causes of digital bullying from the point of view of social media users.	2.45	0.50	638	5.54	0.01**
Females	360		2.65	0.39			
Males	280	Repercussions of digital bullying on social media users.	2.52	0.54	638	4.67	0.01**
Females	360		2.70	0.39			
Males	280	Repercussions of digital bullying on victims’ families.	2.51	0.59	638	1.53	0.44
Females	360		2.58	0.46			
Males	280	The repercussions of digital bullying on society.	2.61	0.61	638	3.47	0.01**
Females	360		2.75	0.38			

*Statistically significant at the level of 0.05 ≥ α; **Statistically significant at the level of 0.01 ≥ α.

The phenomenon of cyberbullying may have a substantial impact on a person’s psychological health, perhaps having a detrimental impact on their mental health. It’s important to remember, however, that cyberbullying is not regarded a direct cause of suicide. Suicidal intents and behavior involve a larger range of components that extend beyond the confines of cyberbullying. The study reveals statistically significant gender variations in respondents’ understanding of the many forms, fundamental causes, and repercussions of cyberbullying. Female participants were shown to have a higher level of awareness of these issues than their male counterparts. Furthermore, respondents with middle-income levels were shown to be more educated about the causes and effects of cyberbullying than those with low- and high-income levels. However, the respondents’ monthly income had no discernible effect on their attitudes about cyberbullying. This research study throws light on the prevalence of gender and wealth disparities in awareness levels, which helps considerably to our knowledge of digital bullying in the Saudi Arabian context.

## 4 Conclusion

The study aimed to identify the repercussions of digital bullying on social media users and used a questionnaire for 640 respondents from students in Saudi universities specified in the study. The study reached the following findings:

1. Regarding the forms of digital bullying, the study found that the majority of respondents agreed statistically with all forms of digital bullying among social media users. The highest percentage was for the item “hostile messages that hurt the feelings of the recipient, such as mockery of appearance, insult, etc.,” while the lowest mean was for impersonation.
2. Regarding the causes of digital bullying, the results found that the majority of respondents agreed statistically on all causes of digital bullying among social media users. The highest percentage was for psychological reasons, followed by social rea-sons, then reasons related to technological development, and in last place economic reasons.
3. As for the repercussions of digital bullying, the results of the study showed that the majority of respondents agreed statistically on all the repercussions of digital bullying on social media users, victims’ families, and society. With regard to the repercussions of digital bullying on social media users, the highest percentage was for the item “low self-esteem and lack of confidence in oneself and others,” while the lowest percentage was for the item “not wanting to practice hobbies.” With regard to the re-percussions of digital bullying on the families of victims, the highest percentage was for “family confusion and lack of knowledge of how to deal with the problem,” and the lowest percentage was for the item “low family productivity.” For the repercussions of digital bullying on society, the highest percentage was for the item “the emergence of bullying and hostile figures against society,” while the lowest percent-age was for the item “the spread of a culture of violence as acceptable solutions to social problems.”
4. The results also showed that female social media users were more aware of the forms and causes of digital bullying among social media users, as well as more aware of the repercussions of digital bullying on both social media users and society, com-pared to males. However, there was no difference in awareness of the repercussions of digital bullying on the families of victims according to gender.
5. The results found that middle-income social media users were more aware of the causes and repercussions of digital bullying among social media users, as well as more aware of the repercussions of digital bullying on victims’ families and society, compared to both low- and high-income monthly earners. However, there was no difference in attitudes towards forms of digital bullying among social media users according to monthly income.

## 5 Recommendations

Based on the findings of the current study, it offers a number of recommendations to reduce the repercussions of digital bullying as follows:

- Awareness campaigns: implement wide public awareness initiatives aimed at reaching a varied population of social media users, regardless of gender. These programmes must prioritize teaching users on the many signs of cyberbullying, its underlying causes, and any possible consequences. People can increase their ability to detect and respond to incidents of cyberbullying by raising their awareness.
- School-based programs: to reduce cyberbullying, educational institutions such as schools and universities should adopt instructional activities. The primary purpose of these activities should be to give children with the knowledge and skills they need to spot and report incidents of cyberbullying. Furthermore, it is critical to emphasize the need of maintaining a secure digital environment for everyone who uses online platforms.
- Psychological support: people who have been the victims of cyber bullying should seek counselling and psychological help. These programmes can assist people in coping with the emotional and psychological consequences of cyberbullying, such as poor self-esteem and confidence.
- Parental involvement: encourage parents to become involved in their children’s online activity. To adequately assist their children if they are victims of cyberbullying, parents must get extensively informed on the indicators of cyberbullying and obtain the information and abilities required to do so. It is critical to promote effective communication between parents and children.
- Social media platforms: collaborate with social media platforms to improve their reporting and moderation functions. It is vital that platforms respond swiftly by taking appropriate action against abusers and that users have simple access to cyberbullying reporting tools. Stricter regulations aimed towards combating cyberbullying can successfully deter future perpetrators.
- Research and monitoring: given the continuing changes in the online world, it is vital to pursue more study on the dynamic nature of cyberbullying. Anti-bullying activities must be reviewed and evaluated on a regular basis to guarantee their continued relevance and efficacy.
- Community support: to help victims and their families, it is essential to establish support networks throughout localities. These networks have the ability to provide advice, emotional support, and access to resources that can help people deal with the challenges brought on by cyberbullying.
- Curriculum integration: include online safety, excellent digital manners, and responsible internet usage training modules in the entire curriculum framework. We can help children acquire a more complete awareness of safe online behavior by teaching them these skills at a young age.
- Legal measures: the inquiry focuses on the implementation of legislation regulations meant to promote accountability for persons who participate in extreme incidents of internet bullying. Legislation might involve the development of legal frameworks that designate cyberbullying as an actionable offence, punishable by warnings, fines, or other legal ramifications.
- Longitudinal research: encourage the conduct of long-term research to analyse the long-term impacts of cyberbullying on people and society. Gaining a full grasp of the long-term repercussions of cyberbullying can assist in the development of more effective preventative and intervention techniques.

## Data availability statement

The raw data supporting the conclusions of this article will be made available by the authors, without undue reservation.

## Ethics statement

The studies involving humans were approved by International Review Board (IRB) Statement: IRB registration number with KACST, KSA: HAP-01-R-059. The studies were conducted in accordance with the local legislation and institutional requirements. The participants provided their written informed consent to participate in this study.

## Author contributions

GA-T: Methodology, Project administration, Validation, Writing – original draft, Writing – review & editing. HA-S: Methodology, Supervision, Writing – review & editing, Data collected by a team.
