# Supplementary material for: The repercussions of digital bullying on social media users
Source: Front Psychol. 2023 Nov 21;14:1280757. doi: 10.3389/fpsyg.2023.1280757 (PMC10699140; doi:10.3389/fpsyg.2023.1280757)
Supplement: Supplementary file 1 [file Data_Sheet_1.docx]

Supplementary Material

# Supplementary Table 1

Table 4. Chi-Square test results to examine the differences between the frequencies of respondents’ responses to the statements of the third axis (the repercussions of digital bullying among social media users).

| # | The Statement | Agree | | Partially Agree | | Disagree | | Average | K^2^ | Rank |
| --- | --- | --- | --- | --- | --- | --- | --- | --- | --- | --- |
|  |  | Freq. | Perc. | Freq. | Perc. | Freq. | Perc. |  |  |  |
| The repercussions of digital bullying on social media users | | | | | | | | | | |
| 1  2 | Mental illnesses such as depression, fear, anxiety, eating and sleeping disorders | 532 | 83.13 | 56 | 8.75 | 52 | 8.13 | 2.75 | 714.06** | 2 |
|  | Exposure to physical illnesses | 348 | 54.38 | 168 | 26.25 | 124 | 19.38 | 2.35 | 132.05** | 10 |
| 3 | A person's feeling of isolation, loneliness, and introversion | 528 | 82.50 | 56 | 8.75 | 56 | 8.75 | 2.74 | 696.21** | 3 |
| 4 | Low self-esteem and lack of confidence in oneself and others | 564 | 88.13 | 40 | 6.25 | 36 | 5.63 | 2.83 | 864.66** | 1 |
| 5 | Suicidal thoughts and self-harm | 464 | 72.50 | 92 | 14.38 | 84 | 13.13 | 2.59 | 441.96** | 7 |
| 6 | Resorting to violence to defend himself | 448 | 70.00 | 132 | 20.63 | 60 | 9.38 | 2.61 | 399.36** | 6 |
| 7 | Low school level and productivity | 496 | 77.50 | 92 | 14.38 | 52 | 8.13 | 2.69 | 565.56** | 5 |
| 8 | Difficulty social adaptation | 516 | 80.63 | 76 | 11.88 | 48 | 7.50 | 2.73 | 645.96** | 4 |
| 9 | Getting into constant quarrels | 420 | 65.63 | 132 | 20.63 | 88 | 13.75 | 2.52 | 304.85** | 8 |
| 10 | Unwillingness to practice hobbies | 396 | 61.88 | 132 | 20.63 | 112 | 17.50 | 2.44 | 235.55** | 9 |
| The repercussions of digital bullying on families of victims | | | | | | | | | | |
| 1  2 | Feeling like failure because they cannot protect their children | 476 | 74.38 | 92 | 14.38 | 72 | 11.25 | 2.63 | 486.06** | 2 |
|  | The family's preoccupation with the circumstances that the children are going through | 444 | 69.38 | 112 | 17.50 | 84 | 13.13 | 2.56 | 375.96** | 5 |
| 3 | Family relationships with others are affected by fear of bullying for their children | 472 | 73.75 | 64 | 10.00 | 104 | 16.25 | 2.58 | 474.21** | 4 |
| 4 | Low family productivity | 340 | 53.13 | 160 | 25.00 | 140 | 21.88 | 2.31 | 113.75** | 8 |
| 5 | Exposure to stress attacks and psychosomatic problems | 432 | 67.50 | 124 | 19.38 | 84 | 13.13 | 2.54 | 339.96** | 6 |
| 6 | Confusion and lack of family knowledge of how to deal with the problem | 508 | 79.38 | 64 | 10.00 | 68 | 10.63 | 2.69 | 610.56** | 1 |
| 7 | The family gets into problems that cause them harm and anxiety | 468 | 73.13 | 88 | 13.75 | 84 | 13.13 | 2.60 | 456.06** | 3 |
| 8 | Financial costs of seeking professional help for the treatment of the victim | 460 | 71.88 | 104 | 16.25 | 76 | 11.88 | 2.60 | 429.66** | 3 |
| 9 | Stop using social media, pretend to be calm and continue life. | 388 | 60.63 | 140 | 21.88 | 112 | 17.50 | 2.43 | 216.35** | 7 |
| The repercussions of digital bullying on families of society | | | | | | | | | | |
| 1  2 | The spread of a culture of violence as acceptable solutions to social problems | 460 | 71.88 | 64 | 10.00 | 116 | 18.13 | 2.54 | 434.16** | 10 |
|  | The spread of fear and the substantial number of crimes | 444 | 69.38 | 108 | 16.88 | 88 | 13.75 | 2.56 | 375.06** | 9 |
| 3 | Impact on psychological and societal security | 544 | 85.00 | 56 | 8.75 | 40 | 6.25 | 2.79 | 769.41** | 2 |
| 4 | Transcending the values of society and moral laws | 500 | 78.13 | 76 | 11.88 | 64 | 10.00 | 2.68 | 578.16** | 7 |
| 5 | The emergence of bullying and hostile figures against society | 560 | 87.50 | 32 | 5.00 | 48 | 7.50 | 2.80 | 845.61** | 1 |
| 6 | Deterioration in the general behaviors of community members | 540 | 84.38 | 60 | 9.38 | 40 | 6.25 | 2.78 | 751.26** | 3 |
| 7 | Loss of respect for others and absence of a culture of community dialogue | 512 | 80.00 | 72 | 11.25 | 56 | 8.75 | 2.71 | 627.81** | 5 |
| 8 | Engaging in anti-social behaviors such as attacks on public property | 508 | 79.38 | 64 | 10.00 | 68 | 10.63 | 2.69 | 610.56** | 6 |
| 9 | The spread of hatred in society | 536 | 83.75 | 48 | 7.50 | 56 | 8.75 | 2.75 | 732.21** | 4 |
| 10 | Increased violations of security laws and regulations | 480 | 75.00 | 80 | 12.50 | 80 | 12.50 | 2.63 | 500.01** | 8 |

** Statistically significant at the level of 0.01≥ α

# Supplementary Table 2

**All Axes (Overall Questionnaire Reliability Statistics)**

| **Overall Reliability Statistics** | | | | | | | |  |  |  |  |  |  |
| --- | --- | --- | --- | --- | --- | --- | --- | --- | --- | --- | --- | --- | --- |
| Cronbach's Alpha | | | Cronbach's Alpha Based on Standardized Items | | N of Items | | |  |  |  |  |  |  |
| .954 | | | .955 | | 40 | | |  |  |  |  |  |  |
| **Overall Scale Statistics** | | | | | | | | | |  |  |  |  |
| Mean | Variance | | | Std. Deviation | | | N of Items | | |  |  |  |  |
| 101.5097 | 287.278 | | | 16.94928 | | | 40 | | |  |  |  |  |
| **ANOVA with Friedman's Test for the Overall Questionnaire** | | | | | | | | | | | | | |
|  | | | | | | Sum of Squares | | | df | | Mean Square | Friedman's Chi-Square | Sig |
| Between People | | | | | | 5910.748 | | | 823 | | 7.182 |  |  |
| Within People | | Between Items | | | | 615.825^a^ | | | 39 | | 15.790 | 1762.210 | .000 |
|  |  | Residual | | | | 10614.475 | | | 32097 | | .331 |  |  |
|  |  | Total | | | | 11230.300 | | | 32136 | | .349 |  |  |
| Total | | | | | | 17141.048 | | | 32959 | | .520 |  |  |
| Grand Mean = 2.5377 | | | | | | | | | | | | | |
| a. Kendall's coefficient of concordance W = .036. | | | | | | | | | | | | | |

**Axe #1 Causes of digital bullying in educational platforms from the students' point of view**

| **Reliability Statistics** | | | | | |  |
| --- | --- | --- | --- | --- | --- | --- |
| Cronbach's Alpha | | Cronbach's Alpha Based on Standardized Items | | N of Items | |  |
| .583 | | .607 | | 4 | |  |
| **Scale Statistics** | | | | | | |
| Mean | Variance | | Std. Deviation | | N of Items | |
| 10.2233 | 3.430 | | 1.85203 | | 4 | |

| **ANOVA with Friedman's Test** | | | | | | |
| --- | --- | --- | --- | --- | --- | --- |
|  | | Sum of Squares | df | Mean Square | Friedman's Chi-Square | Sig |
| Between People | | 705.728 | 823 | .858 |  |  |
| Within People | Between Items | 81.233^a^ | 3 | 27.078 | 208.307 | .000 |
|  | Residual | 882.767 | 2469 | .358 |  |  |
|  | Total | 964.000 | 2472 | .390 |  |  |
| Total | | 1669.728 | 3295 | .507 |  |  |
| Grand Mean = 2.5558 | | | | | | |
| a. Kendall's coefficient of concordance W = .049. | | | | | | |

**Axe #2: Methods of digital bullying in educational platforms from the students' point of view**

| **Reliability Statistics** | | | | | | | |  |  |  |  |  |  |
| --- | --- | --- | --- | --- | --- | --- | --- | --- | --- | --- | --- | --- | --- |
| Cronbach's Alpha | | | Cronbach's Alpha Based on Standardized Items | | N of Items | | |  |  |  |  |  |  |
| .924 | | | .925 | | 16 | | |  |  |  |  |  |  |
| **Scale Statistics** | | | | | | | | | |  |  |  |  |
| Mean | Variance | | | Std. Deviation | | | N of Items | | |  |  |  |  |
| 39.2500 | 67.536 | | | 8.21806 | | | 16 | | |  |  |  |  |
| **ANOVA with Friedman's Test** | | | | | | | | | | | | | |
|  | | | | | | Sum of Squares | | | df | | Mean Square | Friedman's Chi-Square | Sig |
| Between People | | | | | | 3473.906 | | | 823 | | 4.221 |  |  |
| Within People | | Between Items | | | | 234.531^a^ | | | 15 | | 15.635 | 689.351 | .000 |
|  |  | Residual | | | | 3970.594 | | | 12345 | | .322 |  |  |
|  |  | Total | | | | 4205.125 | | | 12360 | | .340 |  |  |
| Total | | | | | | 7679.031 | | | 13183 | | .582 |  |  |
| Grand Mean = 2.4531 | | | | | | | | | | | | | |
| a. Kendall's coefficient of concordance W = .031. | | | | | | | | | | | | | |

**Axe #3: Methods of digital bullying in educational platforms from the students' point of view**

| **Reliability Statistics** | | | | | | | |  |  |  |  |  |  |
| --- | --- | --- | --- | --- | --- | --- | --- | --- | --- | --- | --- | --- | --- |
| Cronbach's Alpha | | | Cronbach's Alpha Based on Standardized Items | | N of Items | | |  |  |  |  |  |  |
| .927 | | | .929 | | 20 | | |  |  |  |  |  |  |
| **Scale Statistics** | | | | | | | | | |  |  |  |  |
| Mean | Variance | | | Std. Deviation | | | N of Items | | |  |  |  |  |
| 52.0364 | 76.283 | | | 8.73401 | | | 20 | | |  |  |  |  |
| **ANOVA with Friedman's Test** | | | | | | | | | | | | | |
|  | | | | | | Sum of Squares | | | df | | Mean Square | Friedman's Chi-Square | Sig |
| Between People | | | | | | 3139.045 | | | 823 | | 3.814 |  |  |
| Within People | | Between Items | | | | 136.917^a^ | | | 19 | | 7.206 | 477.401 | .000 |
|  |  | Residual | | | | 4353.183 | | | 15637 | | .278 |  |  |
|  |  | Total | | | | 4490.100 | | | 15656 | | .287 |  |  |
| Total | | | | | | 7629.145 | | | 16479 | | .463 |  |  |
| Grand Mean = 2.6018 | | | | | | | | | | | | | |
| a. Kendall's coefficient of concordance W = .018. | | | | | | | | | | | | | |
